# *Plasmodium*-Host-*Plasmodium* interactions

**DOI:** 10.1186/1475-2875-9-S2-I8

**Published:** 2010-10-20

**Authors:** Maria M Mota

**Affiliations:** 1 Instituto de Medicina Molecular, Lisboa, Portugal

## 

*Plasmodium* passage through the skin followed by infection of liver hepatocytes and, later, of blood erythrocytes are natural and sequential steps of *Plasmodium* life cycle in the mammalian host and should not be seen as independent entities. Importantly, in regions of high malaria transmission, infected individuals are constantly exposed to potential re-infection. Mosquito bites transmit liver-tropic sporozoites into subjects who are still infected from a previous mosquito bite. What is the impact of ongoing infection in the establishment of a secondary infection is unknown.

Recently, we have asked the question of what would be the impact of an ongoing blood stage infection on a subsequent liver infection. Using a rodent model of infection, we show that ongoing blood stage infections, above a minimum threshold, impair the growth of subsequently inoculated sporozoites such that they become growth arrested in liver hepatocytes and fail to develop into blood stage parasites. This effect is mediated by the host iron regulatory hormone hepcidin, the synthesis of which is stimulated by blood stage parasites and which, by diverting iron away from hepatocytes, impairs the *Plasmodium* liver stage. We model this phenomenon and show that explains the epidemiological patterns of age-related risk and complexity of malaria infections seen in young children. Indeed, concurrent carriage of different parasite genotypes at low asymptomatic parasitaemias is frequently observed in older semi-immune children but not in the young. The interaction between these two *Plasmodium* stages and their host has thus broad implications on the current global efforts to reduce malaria transmission.

